# The Immediate Effects of Different Types of Tea Consumption on Ocular Biometric and Specular Microscopic Parameters in Healthy Subjects

**DOI:** 10.5152/eurasianjmed.2025.24589

**Published:** 2025-04-07

**Authors:** Feyzahan Uzun, Emre Aydın, Hasan Akgöz, Mehmet Gökhan Aslan, Hüseyin Fındık

**Affiliations:** Department of Ophthalmology, Recep Tayyip Erdoğan University School of Medicine, Rize, Türkiye

**Keywords:** Biometry, black tea, green tea, ocular parameters, specular microscopy, white tea

## Abstract

**Background:**

Tea ranks among the most popular beverages globally. In this study, we assessed the short-term changes in ocular biometric and specular microscopic parameters in healthy subjects following the consumption of different types of tea.

**Methods:**

A total of 144 subjects were randomly assigned to 3 groups (black, green, and white tea groups) in this study. Ocular parameters, including axial length (AL), aqueous depth (AD), lens thickness (LT), and central corneal thickness (CCT), were measured using optic biometry. Endothelial cell density (ECD), coefficient of variation (CV), number of hexagonal cells (A6A), and average cell area (AVG) were evaluated using non-contact specular microscopy before, as well as 1 hour and 4 hours after, consuming a cup of tea (containing 60 mg of caffeine per 100 mL).

**Results:**

The average age of the subjects was 32.9 ± 5.9 years. A significant increase in AD values was observed specifically at the 1st hour following the consumption of black, green, or white tea in healthy subjects. Additionally, black and white tea significantly reduced LT in the 1st hour of the study. The differences in AL and CCT values, as well as in specular microscopy parameters, were not significant among all participants after consuming different types of tea.

**Conclusion:**

Black, green, and white tea induce a significant increase in AD, particularly observed 1 hour after oral intake. Furthermore, a reduction in LT parameters was observed in the black and white tea groups, respectively.

**Main Points**

- Tea is the most commonly consumed drink worldwide.
- An essential aspect of achieving a favorable cataract and refractive surgical outcome involves the ability to accurately conduct preoperative calculations.
- Black, green, and white tea induce a significant increase in AD, particularly observed 1 hour after oral intake. Additionally, black and white tea significantly reduced LT in the 1st hour of the study.
- Tea intake of subjects should have been inquired about before conducting critical ocular measurements.

## Introduction

Tea, an aromatic beverage prepared by pouring hot water over processed leaves of *Camellia sinensis*, is the second most widely consumed drink in the world following plain water. In several studies, caffeinated drinks have been shown to affect ocular tissues by increasing intraocular pressure (IOP), enhancing tear production, and potentially improving blood flow to the retina and optic nerve.^1^ In addition to caffeine, tea contains several bioactive molecules, including flavonoids, polyphenols, alkaloids, minerals, and trace elements.^2^ Previous research has shown that tea exhibits positive effects on various diseases, including malignancies, diabetes mellitus, obesity, inflammatory joint diseases, cardiovascular diseases, and stroke, owing to its notable antioxidant, anti-inflammatory, anticarcinogenic, and neuroprotective features.^3^ Although the exact mechanism of active tea ingredients in ocular tissues is not clear, daily intake of tea has been linked to a reduced frequency of cataracts,^4^ alterations in IOP^5^ and neuroprotective potentials on the retina.^6^

The analysis of anterior segment parameters, such as axial length (AL), aqueous depth (AD), lens thickness (LT), and central corneal thickness (CCT), yields crucial data for comprehending the mechanisms of ocular disorders, conducting risk analysis, and monitoring various diseases. Moreover, these measurements are essential for performing accurate preoperative calculations in cataract and refractive surgeries. An essential aspect of achieving a favorable surgical outcome involves the ability to accurately conduct preoperative calculations. With the continuous evolution of modern cataract and refractive surgery techniques and the introduction of new intraocular lens (IOL) designs, there has been a significant improvement in postoperative refractive results. This improvement raises patients’ expectations for achieving a good visual outcome, which depends on the accurate prediction of the power of the implanted IOL, primarily based on preoperative biometric data.

Specular microscopy is a non-invasive diagnostic technique used to assess the corneal endothelium in vivo, applicable to both healthy individuals and those with diverse corneal conditions. Furthermore, imaging of the endothelium is essential for evaluating the donor cornea prior to keratoplasty and for monitoring postoperative progress thereafter.^7^ The hydrophobic properties of caffeine, the major ingredient in tea, enable its passage across all biological membranes, including the cornea. Previous research has demonstrated that caffeine reduces the total thickness of a developing cornea in animal models.^8^ Caffeine intake alters corneal biomechanics by affecting the elasticity and viscosity of the collagen fibers in the corneal stroma.^9^ Following caffeine consumption, researchers observed a reduction in corneal deformability and ultrastructural changes, leading to ocular aberrations.^9,10^

The effect of tea consumption on ocular tissues has been evaluated in previous research. However, to the best of our knowledge, the association of tea with anterior segment parameters and corneal structure has not been thoroughly investigated before. In this study, our aim was to assess the acute changes that may affect ocular biometric measurements and corneal microstructure following the ingestion of different types of tea.

## Material and Methods

In this prospective observational study, we enrolled 144 healthy subjects from the personnel within our department and randomly assigned to 3 groups: black tea, green tea, and white tea groups. All subjects received information about the study’s objectives, and their verbal consent was obtained. This current study adhered to the principles of the Declaration of Helsinki, and the protocol received approval from the Recep Tayyip Erdoğan University Ethics Committee (Approval no: 40465587-050.01.04-219; Date: 28.08.2020) 

Each participant underwent a comprehensive ophthalmological evaluation, which included assessments of best-corrected visual acuity, slit-lamp examination, Goldmann applanation tonometry, pachymetry, and indirect retinoscopy to rule out any previously undiagnosed ocular conditions. Exclusion criteria encompassed abnormalities of the cornea, lens, or ocular surface, as well as a history of glaucoma, prior ocular surgery or trauma, and refractive errors beyond ±2.0 diopters. Individuals who regularly consumed tea (>1 cup daily) were not eligible for participation. Ocular biometric parameters, including AL, AD, LT, and CCT, were measured using non-contact ocular biometry, Lenstar LS 900 (Haag-Streit, Inc., Koeniz, Switzerland). Parameters such as endothelial cell density (ECD), coefficient of variation (CV), number of hexagonal cells (A6A), and average cell area (AVG) were assessed using non-contact specular microscopy Tomey EM-4000 (Tomey Corporation, Japan). Five measurements were recorded for each eye. After discarding the highest and lowest values, the average of the remaining 3 measurements was used for analysis.

Participants were advised to refrain from consuming beverages containing caffeine or chocolate for 24 hours prior to baseline measurements. All measurements were conducted in a consistently illuminated room, maintaining natural pupil size. Following proper head positioning, patients were instructed to gaze at the blue fixation light to ensure consistent accommodation. Baseline measurements were conducted systematically at 9:00 a.m. by the same proficient technician to reduce diurnal variations. Subsequently, participants were provided with a cup of black, green, or white tea, based on their assigned groups. The caffeine content in a cup of tea consumed by patients was adjusted to 60 mg/100 mL per cup according to the manufacturer’s instructions on the tea packages. Ocular biometric measurements were subsequently repeated at the 1st and 4th hours after tea consumption.

Ocular biometric parameters measured from the right eyes were used for the analyses. Statistical analysis was conducted using IBM SPSS (IBM SPSS Corp.; Armonk, NY, USA) version 25 software. The Kolmogorov-Smirnov test was employed to determine the normal distribution of variables. Continuous quantitative variables with a normal distribution are reported as means ± SDs, while categorical variables are presented as absolute numbers and percentages. Baseline demographics were compared among the 3 groups using *χ*^2^ tests for categorical data and one-way analysis of variance (ANOVA) tests for continuous data. The repeated measures ANOVA test was utilized to compare normally distributed values, and the Friedman test was employed to compare nonparametric measures across 3 time points. The one-way ANOVA test was used to analyze variables with a normal distribution, while the Kruskal–Wallis test was employed for variables without a normal distribution to assess group differences.

## Results

The average age of the subjects was 32.9 ± 5.9, ranging from 20 to 44 years. The study enrolled 144 subjects, and an equal number of participants were randomly assigned to each group (n = 48). Among the 144 subjects, 75 were female (52.08%), and 69 subjects were male (47.92%). No significant difference was observed between the groups concerning baseline demographic characteristics.

The mean ocular biometric parameters and specular microscopy values of the participants at baseline, 1st hour, and 4th hour of different types (black, green, and white) of tea consumption are listed in Tables 1 and 2, respectively. Baseline ocular measurements did not show significant differences among all 3 groups (*P* > .05).

Among the ocular biometry measurements, AD was significantly higher in the 1st hour after consuming all types of tea compared to baseline. By the 4th hour, AD showed a slight reduction and was not significantly different from baseline values. Notably, among green tea consumers, the change in AD values between the 1st and 4th hours was significantly different. Intake of black and white tea caused a significant reduction in LT compared with baseline at 1st hour. There was no significant difference between the baseline and 4th-hour values. In the green tea group, LT did not change throughout the study. Additionally, the differences in AL and CCT values were not significant among all participants following the consumption of different types of tea (*P* < .05).

Among the specular microscopy findings, we did not observe any significant changes in any parameters after ingesting 3 different types of tea at the 1st or 4th hours of the study.

## Discussion

In this study, we noted a significant rise in AD values, specifically at the 1st hour following the consumption of black, green, or white tea in healthy subjects. Additionally, black and white tea significantly reduced LT in the 1st hour of the study.

Accurate measurement of ocular biometric parameters and corneal biomechanics could lead to improved diagnosis and monitoring of ocular diseases, such as glaucoma and ectatic corneal disorders, as well as more precise predictions of surgical outcomes. Tea is among the frequently consumed beverages on a daily basis in many countries. It is crucial to understand how recent tea consumption influences ocular measurements for accurate treatment, monitoring, risk assessment, and preoperative calculations related to eye diseases. Three main types of tea—black, green, and white—are obtained via different fermentation and enzymatic oxidization processes. The active ingredients of tea include caffeine (1%-5%) and polyphenols (5%-27%), such as flavonols, theaflavins, and catechins. Epigallocatechin-3-gallate (EGCG) is the main form.^11^ Caffeine found in tea is a methylxanthine that triggers nervous system activity and functions as an adenosine receptor antagonist at normal physiological concentrations, leading to a pharmacological impact on various organ systems. Despite controversial and inconsistent results, several studies have reported the effect of caffeine on ocular tissues. Caffeine has been observed to provide protection against oxidative damage in various animal models of cataracts, likely increased aqueous secretion in rats, and caused vasoconstriction and IOP elevation in the eye.^1^ There is a scarcity of data in the literature concerning the effects of caffeine on ocular anterior segment parameters. Recent research demonstrated that acute caffeine intake resulted in a temporary increase in IOP, a narrowing of the ACA, and had no effect on CCT, ACD, or ACV.^12^ Uzun et al^13^ reported an increase in AD and ACD, accompanied by a decrease in LT, following the consumption of a cup of coffee, with no changes in CCT. Jimenez et al^9^ showed that corneal biomechanics react to caffeine intake, leading to decreased corneal deformity after consuming caffeine. The impact of caffeine on tear production and dry eye disease has yielded conflicting findings, with Osei et al^14^ proposing that caffeine stimulates lacrimal gland secretion, while Juddy et al^15^ argued that caffeinated beverages significantly reduce tear production and exacerbate dry eye symptoms. They speculated that the mechanism behind this reduction is linked to sympathetic stimulation and diuresis. However, in another study, coffee was not identified as a risk factor for dry eye disease.^16^

Besides caffeine, other active ingredients in tea, including polyphenols, catechins, and flavonoids, have demonstrated beneficial effects against various ocular conditions, such as glaucoma, uveitis, senile macular degeneration, and ocular surface diseases. These effects have been evidenced by clinical studies, as well as experiments conducted on cell cultures and animals.^17^ Epigallocatechin-3-gallate, the most prevalent and biologically active catechin, displays anti-inflammatory and antioxidant properties on the corneal epithelium and may also prevent corneal neovascularization.^18^ Reports also provide evidence that EGCG benefits human lens epithelial cells^19^ and retinal pigment epithelium^20^ *in vitro*. In a clinical study, Chang et al^4^ showed a statistically significant reduction in the incidence of cataracts linked to tea consumption. The effect of tea on dry eye disease is also controversial. Nejabat et al^21^ reported that green tea extract is an efficacious, safe, and well-tolerated local treatment for mild to moderate evaporative dry eye and meibomian gland disease. In contrast, Masmali et al^22^ proposed that green tea might have a detrimental effect on tear film quality. They theorized that the negative effects of green tea on the ocular tear film might stem from its high polyphenol content, which could oxidize and damage the lipid layer. In our study, there were no significant differences in CCT and corneal endothelial parameters among healthy subjects after consuming black, green, or white tea. Additionally, we observed a significant increase in AD values in all groups specifically at the 1st hour following the consumption of black, green, or white tea in healthy subjects. Notably, black and white tea significantly reduced LT in the 1st hour of the study. We hypothesized that the effect of tea on the cornea and anterior segment structures might be due to a combination of ingredients rather than any single ingredient. Given the increasing importance of precise corneal thickness and morphological measurements in assessing various diseases such as refractive surgery, glaucoma, and keratoconus, clinicians should be mindful of these effects. They should inquire about the tea intake of subjects before conducting critical ocular measurements.

There are several limitations to our study. The study had a relatively small participant sample size and did not include a control group. The participants were young and healthy, which limits our ability to observe the effects of tea in elderly individuals with ocular pathological conditions. We only assessed changes after the consumption of a single cup of tea (60 mg caffeine/100 mL). Our study concentrated on measuring various ocular biometric parameters at the 1st and 4th hours after ingestion of different types of tea, without extending the experiment beyond this timeframe. Finally, tea is composed of numerous bioactive molecules, and the effects observed on ocular biometric parameters from tea ingestion may not be solely attributable to any single ingredient, but rather to the combined impact of all components.

In conclusion, black, green, and white teas induce a significant increase in AD, particularly observed 1 hour after oral intake. Additionally, black and white teas significantly reduce LT at the 1st hour of the study. A future study with a larger number of participants could be planned using capsules containing specific bioactive ingredients to isolate and demonstrate the individual effects of each on ocular biometric measurements. Additionally, future investigations should include patients with diverse characteristics and various ocular conditions.

## Figures and Tables

**Table 1.** The Changes in Ocular Biometric Parameters in Healthy Subjects Following the Consumption of 3 Different Types of Tea

	AL Mean ± SD	AD Mean ± SD	LT Mean ± SD	CCT Mean ± SD
Black tea				
Baseline (0)	23.61 ± 0.9	2.90 ± 0.3	3.83 ± 0.35	534 ± 30
1st hour (1)	23.62 ± 0.8	2.96 ± 0.4	3.79 ± 0.4	533 ± 29
4th hour (4)	23.62 ± 0.9	2.91 ± 0.3	3.82 ± 0.32	533 ± 28
*P*				
(0-1)	.6	.002*	.005*	.2
(0-4)	.6	1.0	1.0	1.0
(1-4)	1.0	.15	.01*	1.0
Green tea				
Baseline (0)	23.64 ± 0.98	2.88 ± 0.35	3.85 ± 0.37	534 ± 30.4
1st hour (1)	23.64 ± 0.99	3.0 ± 0.42	3.78 ± 0.36	535 ± 27
4th hour (4)	23.65 ± 1	2.88 ± 0.37	3.84 ± 0.36	533 ± 28
*P*				
(0-1)	1.0	.018*	.31	1.0
(0-4)	.46	1.0	.32	1.0
(1-4)	.7	.003*	.85	.4
White tea				
Baseline (0)	23.51 ± 0.93	2.9 ± 0.26	3.84 ± 0.29	533 ± 23
1st hour (1)	23.5 ± 0.93	3.05 ± 0.25	3.73 ± 0.37	534 ± 22
4th hour (4)	23.52 ± 0.92	2.91 ± 0.28	3.82 ± 0.34	534 ± 23
*P*				
(0-1)	1.0	.03*	.02*	1.0
(0-4)	.29	1.0	1.0	1.0
(1-4)	.5	.1	.02*	1.0

*Bold: statistically significant.

AD, aqueous depth; AL, axial length; CCT, central corneal thickness; LT, lens thickness.

**Table 2.** The Changes in Corneal Endothelial Parameters in Healthy Subjects Following the Consumption of 3 Different Types of Tea

	ECD (Cell #/mm^2^) Mean ± SD	CV Mean ± SD	6A (%) Mean ± SD	AVG (μm^2^) Mean ± SD
Black tea				
Baseline (0)	2658.3 ± 264.2	38.2 ± 4.7	8.1 ± 8.1	385.4 ± 37.1
1st hour (1)	2629.4 ± 251.1	39.1 ± 6.9	46.2 ± 9.2	387.6 ± 41.2
4th hour (4)	2605.4 ± 340.5	39.1 ± 7.2	47.4 ± 9.6	389.4 ± 75.1
*P*				
(0-1)	.12	.15	.06	1.0
(0-4)	.29	.23	.7	1.0
(1-4)	1.0	.9	.9	1.0
Green tea				
Baseline (0)	2646.7 ± 272.5	39.2 ± 4.7	49.8 ± 8.1	382.1 ± 33.4
1st hour (1)	2631.3 ± 280.4	40.2 ± 6.9	48.6 ± 9.2	385.3 ± 33.9
4th hour (4)	2628.6 ± 262.1	40.1 ± 7.2	47.8 ± 9.6	384.6 ± 75.6
*P*				
(0-1)	1.0	1.0	1.0	1.0
(0-4)	1.0	.7	.49	1.0
(1-4)	1.0	.47	1.0	1.0
White tea				
Baseline (0)	2655.79 ± 273.5	39.5 ± 4.7	47.81 ± 8.1	387.1 ± 33.4
1st hour (1)	2613.37 ± 263.4	40.29 ± 6.9	46.65 ± 9.1	388.3 ± 38.9
4th hour (4)	2637.61 ± 291.1	41.16 ± 7.2	45.89 ± 9.6	387.6 ± 75.6
*P*				
(0-1)	.3	1.0	1.0	1.0
(0-4)	1.0	1.0	.5	1.0
(1-4)	.8	1.0	.38	1.0

AVG, average cell area; CV, coefficient of variation; ECD, endothelial cell density; 6A, hexagonal cell ratio.

## Data Availability

The data that support the findings of this study are available on request from the corresponding author.
